# Lung Function Impact from Working in the Pre-Revolution Libyan Quarry Industry

**DOI:** 10.3390/ijerph120505006

**Published:** 2015-05-07

**Authors:** Marwan M. Draid, Khaled M. Ben-Elhaj, Ashraf M. Ali, Kendra K. Schmid, Shawn G. Gibbs

**Affiliations:** 1 Faculty of Veterinary Medicine, Department of Pharmacology, Toxicology & Forensic Medicine, University of Tripoli, Tripoli 13662, Libya; E-Mail: marwan8972@yahoo.com; 2 Faculty of Veterinary Medicine, Department of Physiology, Biochemistry & Animal nutrition, University of Tripoli, Tripoli 13662, Libya; E-Mail: khalijedent@yahoo.com; 3 Division of Zoology, Department of Biological Sciences, School of Basic Sciences, The Libyan Academy, Janzour 72331, Libya; E-Mail: ashraf.ali769@yahoo.com; 4 Department of Biostatistics; College of Public Health, University of Nebraska Medical Center, Omaha, NE 68198, USA; E-Mail: kkschmid@unmc.edu; 5 Department of Environmental, Agricultural & Occupational Health, College of Public Health, University of Nebraska Medical Center, Omaha, NE 68198, USA

**Keywords:** Libya, lung function, occupational health, quarry workers

## Abstract

The purpose of this study was to determine the lung impact from working within the Libyan quarry industry, and if the length of work impacted the degree of degradation. Eighty three workers from eight silica quarries in the Nafusa Mountains of Libya opted to participate. These quarries were working the upper cretaceous geological structure. Eighty-five individuals who lived in Gharyan City with no affiliation to quarry operations participated as controls. Spirometry variables evaluated were Forced Vital Capacity (FVC), Forced Expiratory Volume at 1.0 second (FEV1), FVC/FEV1 and Peak Expiratory Flow (PEF). Control and exposed groups had no differences in terms of height, weight, or smoking status (*p* = 0.18, 0.20, 0.98, respectively). Prior to adjustment for other variables, FVC, FEV1, and PEF are all significantly lower in the exposed group (*p* = 0.003, 0.009, 0.03, respectively). After adjustment for age, height, weight, and smoking status, there remain significant differences between the control and exposed groups for FVC, FEV1, and PEF. This analysis demonstrated that exposure to quarry dust has a detrimental effect on lung function, and that pre-revolution Libyan quarry workers were being exposed. This study shows that any exposure is harmful, as the reduction in lung function was not significantly associated with years of exposure.

## 1. Introduction

Respiratory disease associated with smoke and dust exposure is a global issue that disproportionately impacts developing nations [1,2,3,4]. The quarry industry in Libya is one of the largest industries, and its workers are routinely exposed to dust whose aerodynamic diameter fall within the respirable size range throughout various stages in the quarrying process [2,3]. In Libya, like many other developing nations, rock quarries utilize a great deal of manual labor with minimal personal protective equipment, and rarely is respiratory protection utilized. It has been established that occupational exposure to environments with high dust levels increase that risk of inhaling particles that could have negative respiratory effects [4,5]. This has the potential to lead to lung damage from exposure to the aerosolized dust, particularly in the hot desert environment found within Libya. While the health impacts to workers within the quarry industry has been studied in many different countries, it has been relatively unexplored in Libya [6].

The occupational exposure to quarry dust may cause a number of health effects, including but not limited to the onset of acute or chronic respiratory diseases and respiratory functional deficits. Lung function impairment is one of the most common occupational respiratory problems associated with occupation dust exposures [7,8]. A number of studies have evaluated the impact of dust on lung function, but most of these studies were conducted without considering the duration of the exposure and none of these studies have been performed in Libya [2,3,4,5,6,7,8]. Further clues may lie in the pattern of lung function deficit associated with dust exposure. There is some indication that the loss of Forced Vital Capacity (FVC) relative to Forced expiratory volume measured at one second (FEV1) is greater from dust than from smoking [9,10,11]. Dust exposure has been determined to result in a number of negative health impacts [11].

The purpose of this study was to determine the impacts to lung function from working in the Libyan quarry industry, and if the length of work at a quarry impacted the degree of degradation to lung function.

## 2. Experimental Section 

### 2.1. Study Group

Human Subjects Approval was obtained through the School of Basic Studies in the Libyan Academy (Formerly, the Academy of Graduate Studies) in Janzour, Libya. Eight quarries in the Nafusa Mountains of Libya about 140 km west of Tripoli were the recruitment sites within this study. Approximately 10 workers from each quarry opted to participate for a total exposure sample size of 83 participants. These quarries were working on the upper cretaceous geological structure. The mineral being quarried was silicon dioxide (SiO_2_) or silica, which was primarily used in concrete for building houses.

Control participants were recruited from the nearby Gharyan City, Libya which was in close proximity to the quarries. Eighty-five individuals who lived in Gharyan City but had no affiliation with the quarry operations agreed to participate as controls. A convenience sampling approach was used to recruit quarry workers with all workers approached agreeing to participate. A convenience sampling approach was used, in which office workers from the area around each quarry were recruited for their similarity in age and gender to those participating in the exposure group. All potential control subjects approached agreed to participate. Only those potential control or exposure participants who had undergone abdominal or chest surgery were excluded from the study. All study participants were male.

### 2.2. Questionnaire for Workers

Male data collectors conducted a face to face questionnaire for each worker that included age, weight, height, smoking status, sex and questions about health status. This data was collected from May 2009 to December 2010 just ahead of the Libyan Revolution of 2011, when as a result of the Revolution several planned follow-up visits were not possible as this area saw a great deal of combat, and continues to be volatile. Participants were asked if they smoked, and for how many years. As the smokers in the population predominately rolled their own cigarettes, measures such as pack-years could not be effectively determined.

### 2.3. Measurements

Typically, airflow obstruction may be diagnosed using spirometry alone by demonstrating a lower than predicted FEV1/FVC ratio (as occurs in chronic obstructive pulmonary disease (COPD) and emphysema [10]. This commonly used lung function test measures how much and how quickly air can be moved out of the lungs. The subject breathes into a mouthpiece attached to a recording device (spirometer), which generates the spirogram. Common lung function values measured with spirometry and utilized in this study are: Forced vital capacity (FVC) which measures the amount of air that can be exhaled with force after inhaling as deeply as possible, Forced expiratory volume measured at one second (FEV1) which measures the amount of air that can be exhaled with force in one breath measured at one second, and peak expiratory flow (PEF) which measures how quickly you can exhale [10]. Spirometry measurements were collected according to the equipment manufacturer’s recommendation with a flow-volume spirometry device (Spiroanalyzer ST-95, Fukuda Sangyo, Antipolo City, Philippines) with the participant seated. Each subject performed three forced expiratory maneuvers and inspiratory spirograms were recorded in conjunction with expiratory spirograms whenever feasible. The spirometry variables evaluated in this study were Forced Vital Capacity (FVC), Forced Expiratory Volume at 1.0 second (FEV1), FVC/FEV1 and Peak Expiratory Flow (PEF). Values were reported based on the best of three technically acceptable tests [10].

### 2.4. Statistical Analysis

Descriptive statistics were computed as mean and standard deviation for continuous variables and number and percent for categorical variables and compared between groups using t-tests and chi-square tests, respectively. Linear models were used to determine if there were significant differences in the outcome variables (FVC, FEV1, PEF, FEV1/FVC) between groups after adjusting for age, height, weight, and smoking status. Differences in means were calculated to compare groups with significant differences. Relationships between outcome measures for the exposed group and years working in quarry were investigated with Pearson’s correlation. Linear models were used to determine if there was a significant effect of years working in the quarry on the outcome variables (FVC, FEV1, PEF, FEV1/FVC) after adjusting for age, height, weight, and smoking status. Data was analyzed using SAS version 9.3 and *p* values < 0.05 will be considered statistically significant.

## 3. Results and Discussion

Our study of pre-revolution Libya found that exposure to quarry dust has a detrimental effect on lung function; however, we were did not find reduction in lung function associated with years of exposure. While this is the first time this has been demonstrated in quarry workers in Libya, the findings concur with a number of other studies.

Group characteristics and summary measures including mean, standard deviation, and 25th and 75th percentiles are reported in Table 1. There were no differences between control and exposed groups in terms of height, weight, or smoking status (*p* = 0.18, 0.20, 0.98, respectively); age was marginally significant (*p* = 0.05) with the mean age in the control group slightly higher than that of the exposed group. Prior to adjustment for other variables, FVC, FEV1, and PEF are all significantly lower in the exposed group (*p* = 0.003, 0.009, 0.03, respectively). After adjustment for age, height, weight, and smoking status, there remain significant differences between the control and exposed groups for FVC, FEV1, and PEF. After adjustment, the mean for FVC is 0.27 (Standard Error of the Mean (SEM) 0.09, *p* = 0.003, 95% CI: 0.10 to 0.44) lower in the exposed group than the control group. Similarly, FEV1 averages 0.21 (SEM 0.07, *p* = 0.003, 95% CI: 0.07 to 0.35) lower for exposed than controls and PEF averages 40.7 (SEM 19.2, *p* = 0.04, 95% CI: 2.8 to 78.5) lower.

A sub analysis was conducted on only individuals who worked in the quarry. FVC, FEV1, and PEF all showed slight negative correlations with number of years working in the quarry, however, none of these were statistically significant. Age and years of work had a moderate correlation of 0.43 (*p* < 0.001). After adjustment for age, height, weight, and smoking status, there was no significant effect of years worked on any of the outcomes (all *p* > 0.4). Due to the correlation between age and years worked, age was removed from the models and still, no significant effect was found from years worked in the quarry on any outcome measure.

The researchers had expected to find a reduction in lung function associated with years of exposure but our study did not find such an association; there are several possible reasons our study did not detect this result. It is possible that our study was not large enough to detect a correlation between reduction in lung function and years of exposure. Additionally, only about 14% of our study participants had more than ten years of exposure working in the quarry with 67% of participants having worked in the quarry for less than five years, and 18% having worked in the exposure for between six and ten years. Perhaps if more participants had worked in the quarry for greater than ten years we would have been able to detect this effect, but even so, it seems even short exposure resulted in a reduction in lung function. This is one of the limitations or biases that may have impacted this study. It is also possible that the convenience sampling technique resulted in a bias that may have been reduced by a true randomized sampling scheme. Additionally, the lack of quantitative sampling is also another bias. This study utilized employment at the quarry as a proxy for the dust exposure, and no quantitative data on dust exposure was collected from either the exposure or control participants. It is a weakness of the study that neither personal nor area samplers were used to quantify dust exposure. While the study would be stronger with this quantitative exposure data, the resources were not available to collect this quantitative data.

**Table 1:** Summary measures of control and exposed groups. Mean (Standard deviation) (25th percentile, 75th percentile).

Variable	Control Group	Exposed Group	t-test or Chi-Square test *p*-Value
Age (years)	35.2 (8.8) (31.0, 40.0)	32.5 (9.4) (25.0, 38.0)	0.05 *
Height (cm)	172.8 (5.5) (170.0, 176.0)	171.6 (6.5) (167.0, 175.0)	0.18
Weight (kg)	77.6 (14.2) (69.0, 85.0)	74.7 (14.1) (64.0, 85.0)	0.20
FVC (liters)	3.8 (0.5) (3.5, 4.2)	3.6 (0.7) (3.1, 4.1)	0.003 *
FEV1 (liters)	3.4 (0.5) (3.1, 3.6)	3.1 (0.5) (2.8, 3.4)	0.009 *
PEF (liters/min)	466.2 (150.4) (450.0, 540.0)	422.7 (100.0) (350.0, 490.0)	0.03 *
FEV1/FVC (%)	87.6 (7.5) (82.4, 93.8)	89.1 (8.7) (84.1, 95.8)	0.24
Smoking (%)	42.4%	42.2%	0.98
Exposure (years)	NA	5.0 (4.7) (2.0, 8.0)	NA

* Indicates statistical significant difference.

A number of other studies have also found decreased lung function of quarry workers, and other occupations with heavy dust exposure. An Italian study found that pottery workers exposed to silica had reduced lung function according to spiromectric data [12]. While there are differences in the occupational environments between Italy and Libya, the reported findings of decreased lung function associated with dust exposure is similar. A study within Nigeria examined over 400 workers at a quarry and found reduced lung function throughout the study population’s age range of 10 to 60 years of age [13]. These studies, like ours, found significant decreases in both FEV1 and FVC measurements within their exposed population of quarry workers.

The results of the exposures observed in our study and other studies regarding silica dust exposure from quarry work are all preventable. Occupational exposure to dusts has been demonstrated as a contributor to pulmonary diseases, such as asthma and bronchitis [14]. These and other negative health effects of silica dust exposure are widely recognized, but these exposures continue to occur globally. While workplace controls are being implemented in developed nations, little is done in the developing countries [15]. Libya faces many changes and challenges as it continues in the Post-Qadhafi era that are more pressing than the long-term effects of silica exposure on quarry workers. However, as it navigates through the redevelopment of its infrastructure there is an opportunity to put a greater emphasis on occupational health and safety, including the health of quarry workers [16]. Western nations seem to have an interest in cooperation and development with Libya, which could include the development of occupational health culture as part of the infrastructure redevelopment that could make it a model for African nations. Taking this stance would have long lasting implications throughout many industries, including the quarry industry, for years to come that would have a beneficial impact on the health and wellbeing of all Libyan workers.

## 4. Conclusions 

This analysis has demonstrated that exposure to quarry dust has a detrimental effect on lung function. The exposed group in this study has significantly lower lung function than their age and gender matched counterparts. Three of four outcome variables studied show reduced lung function for those who have been exposed as compared to controls, and these effects are independent of age, height, weight, and smoking status. Additionally, this study shows that any exposure is harmful, as the reduction in lung function was not significantly associated with years of exposure.
